# Hepatoprotective effect of ethanolic extract of *Curcuma longa* on thioacetamide induced liver cirrhosis in rats

**DOI:** 10.1186/1472-6882-13-56

**Published:** 2013-03-05

**Authors:** Suzy M Salama, Mahmood Ameen Abdulla, Ahmed S AlRashdi, Salmah Ismail, Salim S Alkiyumi, Shahram Golbabapour

**Affiliations:** 1 Department of Molecular Medicine, Faculty of Medicine, University of Malaya, 50603, Kuala Lumpur, Malaysia; 2 Institute of Biological science, Faculty of Science, University of Malaya, 50603, Kuala Lumpur, Malaysia

**Keywords:** *Curcuma longa*, Antioxidant enzymes, Cytochrome P450 2E1 (CYP2E1), Histology, Oxidative stress, Immunohistochemistry

## Abstract

**Background:**

Hepatology research has focused on developing traditional therapies as pharmacological medicines to treat liver cirrhosis. Thus, this study evaluated mechanisms of the hepatoprotective activity of *Curcuma longa* rhizome ethanolic extract (CLRE) on thioacetamide-induced liver cirrhosis in rats.

**Methods:**

The hepatoprotective effect of CLRE was measured in a rat model of thioacetamide-induced liver cirrhosis over 8 weeks. Hepatic cytochrome P450 2E1 and serum levels of TGF-β1 and TNF-α were evaluated. Oxidative stress was measured by malondialdehyde, urinary 8-hydroxyguanosine and nitrotyrosine levels. The protective activity of CLRE free-radical scavenging mechanisms were evaluated through antioxidant enzymes. Protein expression of pro-apoptotic Bax and anti-apoptotic Bcl-2 proteins in animal blood sera was studied and confirmed by immunohistochemistry of Bax, Bcl2 proteins and proliferating cell nuclear antigen.

**Results:**

Histopathology, immunohistochemistry and liver biochemistry were significantly lower in the *Curcuma longa*-treated groups compared with controls. CLRE induced apoptosis, inhibited hepatocytes proliferation but had no effect on hepatic CYP2E1 levels.

**Conclusion:**

The progression of liver cirrhosis could be inhibited by the antioxidant and anti-inflammatory activities of CLRE and the normal status of the liver could be preserved.

## Background

Cirrhosis is the damage of liver cells and their gradual replacement with scar tissue that impairs blood flow through the liver causing hepatocyte death and loss of liver function [1]. Hepatic fibrosis occurs in response to liver damage and regenerates apoptotic cells after repeated injury [2]. This inflammatory response is accompanied by limited deposition of extra cellular matrix (ECM), so that if the regeneration of dying cells fails during persistent liver injury, hepatocytes are replaced by abundant ECM, including fibrillar collagen, depending on the origin of injury [3]. Treatment options for common liver disease such as cirrhosis, fatty liver and chronic hepatitis are problematic. The effectiveness of treatments such as interferons, colchicines, penicillamine and corticosteroids are inconsistent at best and the incidence of side-effects profound [4]. Because of the role of oxidative stress in liver cirrhosis, antioxidants have been proposed as a treatment for cirrhosis [5]. Several studies have demonstrated the protective effects of antioxidants against induced liver injury by reducing oxidative stress in cells [6,7]. A number of herbals show promising activity, including Silymarin for liver cirrhosis, glycyrrhizin for chronic viral hepatitis, and herbal combinations from China and Japan that have been proven for treatment of liver diseases [8]. Silymarin, a reference drug, is a flavonolignan from ″milk thistle″ *Silybum marianum,* and widely used for the treatment of hepatitis and liver cirrhosis [9].

*Curcuma longa* is a rhizomatous perennial herb that belongs to the family *Zingiberaceae*, native to South Asia and is commonly known as turmeric. In Malaysia**,** commonly known as Kunyit, turmeric plant is a popular ingredient for preparing culinary dishes. In addition, it is used as herbal remedy due to the prevalent belief that the plant has medical properties. In folk medicine, the rhizome juice from *C. longa* is used in the treatment of many diseases such as anthelmintic, asthma, gonorrhea and urinary, and its essential oil is used in the treatment of carminative, stomachic and tonic [10]. In traditional medicine, several plants and herbs have been used experimentally to treat liver disorders, including liver cirrhosis, [11,12]. *C. longa* possesses antioxidant [13], anti-tumor [14], antimicrobial [15], anti-inflammatory [16], wound healing [17], and gastroprotective activities [18]. The previous studies have also shown that the aqueous extract of *C. longa* has hepatoprotective activity against carbon tetrachloride toxicity [19]. In this study, we assessed the hepatoprotective effect of the ethanolic extract of *C. longa* rhizomes against TAA-induced liver cirrhosis in Sprague Dawley rats.

## Methods

### Preparation of CLRE

*C. longa* rhizomes were obtained from Ethno Company, Kuala Lumpur, Malaysia and identified by comparison with the voucher specimen (KLU41829) deposited at the Herbarium of Rimba Ilmu, Institute of Biological Sciences, University of Malaya, Kula Lumpur, Malaysia The rhizomes were cleaned, dried, ground, weighed, and homogenized in 95% ethanol at a ratio of 1:10 of plant to ethanol and left to soak for 3 days at 25°C with occasional shaking and stirring. The mixture was then filtered and the resulting liquid was concentrated under reduced pressure at 45°C in an EYELA rotary evaporator to yield a dark gummy-yellow extract (7%, w/w). The concentrated extract was then kept in the incubator at 45°C for 3 days to evaporate the ethanol residue yielding the crude rhizome extract. Extracts were then dissolved in 10% Tween-20 before being orally administrated to animals in concentrations of 250 and 500 mg/kg body weight (5ml/kg body weight).

### Total phenol content (TPC) of CLRE

The Total Phenol content (TPC) of the CLRE extract was determined by the Folin Denis calorimetric method using Folin-Ciocalteau reagent (Merck, Darmstadt, Germany) in gallic acid equivalent in mg (GAE/mg extract) [20]. CLRE (1 mg) was first dissolved in 1 mL dimethyl sulfoxide (DMSO). Next, 20 μL of the extract was added into 100 μL of Folin-Ciocalteau reagent, and the resulting mixture was incubated in the dark for 3 min. Then, 100 μL of sodium carbonate (1 g/10 mL) solution was added to the mixture, and mixed thoroughly. The final mixture was kept in the dark for 1 h and its absorbance (750 nm wavelength) was read by an ELISA reader (UV 1601 spectrophotometer, Shimadzu, Japan). All procedures were carried out in triplicate. Linear standard curves were produced by serial dilution of gallic acid (1 mg/mL DMSO) and the absorbance was read at 750 nm.

### Ferric reducing anti-oxidant power of CLRE

The ferric reducing anti-oxidant power (FRAP) of CLRE was assayed according to the previously described method [21] with slight modification. FRAP reagent was prepared by adding 300 mM acetate buffer (3.1 mg sodium acetate/mL, pH 3.6) to 10 mM 2,4,6-tripyridyl-S-triazine (TPTZ) solution (Merck, USA) and 20 mM FeCl_3_.H_2_O (5.4 mg/mL). Ten μL of 1 mg/mL of CLRE (equivalent to 500 mg/kg dose administrated daily to animals) and the standards gallic acid, quercetin, ascorbic acid, retin, trolox and 2,6-di-tert-butyl-4 methyl phenyl (BHT) were each sampled with 10 μL of 0.1 mg/mL Silymarin (equivalent to 50 mg/kg dose administrated daily to animals) and added to 290 μL of TPTZ reagent in triplicate wells. Absorbance was read at 593 nm using an ELISA reader (Shimadzu, Japan) every 4 min for 2 h.

### Experimental animals

Sixty-six healthy *Sprague Dawley* rats (180-250 g) were used in the experiments. All rats were kept in wire-bottomed cages at 25 ± 2°C, given tap water and standard pellet diet and exposed to a 12 h:12 h light–dark cycle at 50–60% humidity in an animal room. Throughout the experiments, all animals received human care according to the criteria outlined in the “Guide for the Care and Use of Laboratory Animals” prepared by the National Academy of Sciences and published by the national Institute of health. The study was approved by the Ethics Committee for Animal Experimentation, Faculty of Medicine, University of Malaya, Malaysia PM/28/08/2009/MAA.

### Acute toxicity study

Eighteen males and eighteen females healthy rats were assigned equally into 3 groups of 6 rats: vehicle (receiving 10% Tween-20 w/v, 5 mL/kg); or treated with 2 g/kg or 5 g/kg of CLRE preparation, respectively. The animals were fasted overnight but water prior to dosing. Food was withheld for a further 3-4 h after dosing. The animals were observed for 30 min and at 2, 4, 8, 24 and 48 h after administration for the onset of clinical or toxicological symptoms. The animals were sacrificed on the 15^th^ day. Histological and serum biochemical parameters were determined using standard methods [22].

### Induction of liver cirrhosis in rats

Thioacetamide (TAA, CH_3_-C(S)NH_2_) is a hepatotoxin and hepatocarcinogenic when administered in the diet of experimental animals, and is widely used as a model of acute and chronic liver disease [23] Briefly, after administration of TAA in the diet, it is converted to TAA-S-oxide (TASO) by hepatic microsomal cytochrome P450 2E1 (CYP2E1), then transformed to toxic thioacetamide S-dioxide (TASO_2_) [24]. TASO_2_ damages biomolecules of the liver leading to cirrhosis [25].

Male animals were randomly divided into 5 groups of 6 rats. Rats of Group 1 (normal control group) were orally administrated with 10% Tween-20 (5 mL/kg) daily and intraperitoneally (ip) injected with sterile distilled water (1 mg/kg) thrice weekly. Groups 2–5 were administered with TAA by intraperitoneal injection (200 mg/kg/mL) three times a week to induce liver cirrhosis. Constant exposure of this concentration of TAA induces pathological changes in the liver comparable to the etiology of cirrhosis in humans [26]. The stock solution was prepared (5 g/L) by dissolving TAA crystals (Sigma-Aldrich, USA) in sterile distilled water and stirred till completely dissolved [27]. Rats of Group 2 (cirrhosis control group) were orally administrated with 10% Tween-20 (5 mL/kg) daily. Rats of Group 3 (Silymarin-treated group) were orally administrated with Silymarin (50 mg/kg) daily. Silymarin (International Laboratory, USA) was properly dissolved in 10% Tween-20 and used as a standard drug. Rats of Groups 4 and 5 (treatment groups) were orally administrated with CLRE at daily doses of 250 mg/kg and 500 mg/kg, respectively. The treatment procedure was considered an 8-week period due to the preventive nature of the experiment (Silymarin and CLRE), protecting the liver from further damage. At the end of the 8 weeks, the rats were fasted for 24 h after the last treatment and perfused under ketamine (30 mg/kg, 100 mg/mL) and xylazil (3 mg/kg, 100 mg/mL) anesthesia [28]. Blood was withdrawn through the jugular vein and collected for prothrombin time ratio evaluation, biochemical examinations, cytokines and apoptotic proteins assessment. Liver tissues were excised, washed with ice cold normal saline, blotted on filter paper and weighed. The tissues were examined thoroughly for gross cirrhosis. They were prepared for evaluation of the oxidative damages and histopathology assessment. Liver tissues were homogenized (10% w/v) in 50 mM cold potassium phosphate buffer (pH 7.4) using a Teflon homogenizer (Polytron, Heidolph RZR 1, Germany). Then the tissue homogenates were centrifuged at 3500 rpm for 15 min at 4°C in a centrifuge (Heraeus, Germany). The supernatant of each sample was collected and frozen in aliquots for later use.

### Biochemical analysis

Blood samples from animals were collected in sodium citrate tubes for determining prothrombin time or in gel-activated tubes for the assessment of specific liver markers. The gel-activated tubes were allowed to clot, then centrifuged at 3400 rpm for 10 min at 4°C. The serum samples were collected for measuring liver markers, alkaline phosphatase (AP), alanine aminotransferase (ALT), aspartate aminotransferase (AST), total protein, albumen and bilirubin. The markers were assayed with a spectrophotometer at Central Diagnostic Laboratory of the Medical Center of University Malaya.

### Assessment of hepatic CYP2E1 levels

The level of CYP2E1 in the liver tissue homogenate of all rats was evaluated by following the instructions of a sandwich enzyme immunoassay (Uscn Life Science, China). Briefly, 100 μL of the sample was incubated with pre-coated capture antibody specific to CYP2E1 in a 96-well plate for 2 h at 37°C. After mild rinsing, the sample was incubated for 1 h at 37°C with 100 μL of biotin-conjugated secondary antibody followed by three times washing by 350 μL washing buffer. Streptavidin-Horse radish peroxidase (HRP) was added (100 μl) to the sample and incubated for 30 min at 37°C followed by 5 repeated washes. Tetramethylbenzidine (TMB) was added (90 μL) to the sample as a colorimetric reagent and incubated for 20 min, stopped by H_2_SO_4_ (50 μL) and the absorbance was read at 450 nm.

### Evaluation of oxidative stress markers

#### Urine 8-OH-dG

8-hydroxy-2-deoxyguanosine (8-OH-dG) is a product of DNA oxidative damage through reactive oxygen species (ROS) and serves as an established oxidative stress marker [29]. To evaluate the DNA oxidative damage, urine samples from all animals were collected 24 h before sacrifice and stored (-80°C). The levels of 8-OH-dG were measure according to the instructions of the manufacturer (Genox KOG-HS10E, USA). In brief, in a 96-well microtiter plate, pre-coated with monoclonal antibody specific for 8-OH-dG, a urine sample (50 μL) was incubated at 4°C overnight. The plate was then washed (5 times) with concentrated buffered saline (pH=7.4). 100 μL of biotinylated secondary antibody was added to the sample and incubated for 1 h at room temperature followed by a 3-time rinsing. The chromatic solution tetramethylbenzidine (TMB) was added (100 μL) and incubated at room temperature for 15 min in dark. The reaction terminating solution (1M phosphoric acid) was added (100 μL) and the plate was read with a spectrophotometer at 450 nm.

#### Hepatic nitrotyrosine

Nitrotyrosine, a marker for protein oxidation [30] was assessed in liver tissue homogenate of all animals by ELISA according to the manufacturer protocol (MyBiosource, USA). Succinctly, 100 μL of the sample was incubated with monoclonal Nitrotyrosine-HRP conjugate in a microtiter plate. After 1 h of incubation, the plate was washed 5 times by 350 μL wash solution. Then substrate specific to HRP enzyme was added (100 μL) to the sample followed by 50 μL stop solution and the intensity of the produced colour was measured with a spectrophotometer at 450 nm.

#### Hepatic malondialdehyde

Malondialdehyde (MDA) levels were measured in the liver tissue homogenate of all experimental groups as a measure for lipid peroxidation using thiobarbituric acid according to the manufacturer’s instructions (Cayman, Sigma). Ready to use SDS solution was added (100 μL) to the samples/standard (100 μL). Then 4 mL of the colour reagent were added to the mixture. Samples and standard solutions vials were immersed in boiling water for 1 h. The reaction was stopped when they were incubation in an ice bath for 10 min. All vials were then centrifuged at 1600 × g for 10 min at 4°C. A set of duplicated samples or standards were loaded into 96-well plate, and their absorbance were read at 532 nm by a spectrophotometer.

#### Antioxidant enzyme assessment

Superoxide dismutase (SOD) and catalase (CAT) enzymes were measured for each liver tissue homogenate (Cayman, USA). SOD activity was evaluated by tetrazolium salt detecting superoxide radicals produced by the action of xanthine oxidase on hypxanthine. Bovine erythrocyte SOD was used to represent SOD standard curve. 200 μL of tetrazolium salt solution was added to 10 μL standards or samples followed by fast addition of 20 μL of xanthine oxidase to initiate the reaction. The plate was covered and incubated for 20 min on a plate shaker (Barnstead Dubuque, USA) and the reading of the spectrophotometer was recorded at 450 nm. CAT activity was evaluated by the chromagen, 4-amino-3 hydrazino-5-mercapto-1,2,4-triazole which measures the formaldehyde produced by the reaction of CAT enzyme with methanol in the presence of H_2_O_2._ The standard curve was obtained by catalase formaldehyde standard. Assay buffer (100 μL) and methanol (30 μL) were added to the standards or samples (20 μL) and the reaction was initiated when 20 μL of H_2_O_2_ was added. The plate was incubated in dark at room temperature for 20 min. Then Potassium Phosphate buffer (30 μL) was added to terminate the reaction. Chromagen was added (30 μL) and the plate was incubated on a shaker at room temperature in the dark. After 10 min, catalase potassium periodate was added (10 μL) and the plate was covered and left on the shaker at room temperature for 5 min. The absorbance was measure with a spectrophotometer at 540 nm.

#### Assessment of cytokines

Blood samples from each group was centrifuged at 3500 rpm and the sera were stored (-80°C) in aliquots for assessment of transforming growth factor-beta (TGF-β), a fibrogenesis-driving cytokine, and tumornecrosisfactor-alpha (TNF-α), according to the manufacturers’ instructions (Abnova, USA). Briefly, the captured antibody was diluted with the sample buffer provided and added onto 96-plate pre-coated with anti-rat antibody specific to TGF-β or TNF-α. After the recommended incubation period, biotinylated anti-rat specific antibody was added and left incubated at 37°C in the dark. Then the samples and the standards were incubated with streptavidin-HRP conjugate which was washed with the washing buffer. The wells were then incubated with the colorimetric reagent TMB and the reaction was stopped so that to read the absorbance at 450 nm. The concentrations were calculated by the optical density measurements from the obtained standard curve.

#### Pro-apoptotic Bax and anti-apoptotic Bcl-2 assessment

Rat Bax ELISA kit and Rat Bcl-2 ELISA kit (Uscn Life Science, China) were used to evaluate the expression of Bax and Bcl-2 proteins in the rat sera. Prior to use, sera samples stored at -80°C were warmed up in 37°C bath and then protein concentration in the samples was measured. The absorbance was read at 450 nm and the concentration ratio of Bax/ Bcl-2 was then calculated accordingly.

#### Histopathological analysis

Liver samples were fixed in 10% buffered formaldehyde, processed by an automated tissue processing machine followed by paraffin wax embedding. Sections (5 μm in thickness) were prepared and stained with hematoxylin and eosin (H&E) for histopathological examination of the liver tissue. Staining with Masson’s Trichrome (Sigma, USA) was used as a marker of fibrosis to assess the degree of fibrosis by identifying collagen fibers in liver tissues. All the slides were examined under a light microscope and images were captured with a Nikon microscope (Y-THS, Japan).

#### Immunohistochemistry

Liver tissue sections were heated at 60°C for 25 min in an oven (Venticell, MMM, Einrichtungen. Germany) and then deparaffinized in xylene and rehydrated using graded alcohol. The process of antigen retrieval was performed in 10 mM sodium citrate buffer boiled in a microwave. Immunohistochemistry staining steps were performed following the manufacturer’s instructions (DakoCytomation, USA). In brief, endogenous peroxidase was blocked using 0.03% hydrogen peroxide sodium azide for 5 min. Tissue sections were washed gently with wash buffer and then incubated with Bcl-2–associated X protein (Bax) (1:500), Proliferating Cell Nuclear Antigen (PCNA) (1:200) and anti-apoptotic protein Bcl2 (1:50) biotinylated primary antibodies for 15 min. Sections were gently washed with wash buffer and kept in the buffer bath in a humid chamber. A sufficient amount of streptavidin-HRP was then added and incubated for 15 min followed by washing. Diaminobenzidine-substrate chromagen was added to the sections and incubated for over 7 min followed by washing and counterstaining with hematoxylin for 5 sec. The sections were then dipped in weak ammonia (0.037 M/L) 10 times, washed and cover slipped. Positive antigens stained brown under light microscopy.

#### Statistical analysis

Statistical analysis of the results was performed using one-way ANOVA (Tukey Post-Hoc Test analysis) using SPSS version 18 (SPSS Inc, USA). All values were reported as mean ± SEM and a value of *P<*0.05 was considered statistically.

## Results

### TPC and FRAP results

**Figure 1:**
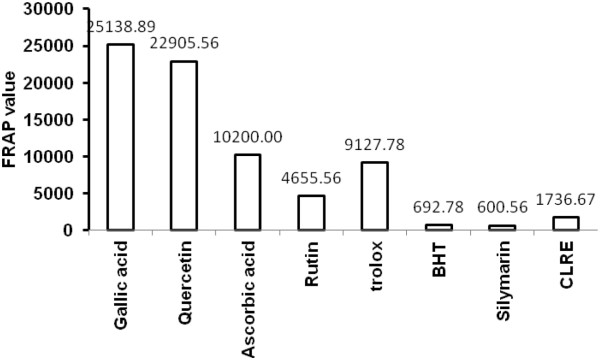
**Antioxidant activity of the CLRE compared with the standards: gallic acid, quercetin, ascorbic acid, rutin, trolox, BHT and the standard drug Silymarin.** Values were expressed as mean ± SEM.

The TPC of the CLRE was 517.54 ± 0.049 mg GAE/mg extract, while the calibration curve equation was y = 0.15× + 0.0557, R^2^ = 0.9867. The FRAP of 1 mg/mL of CLRE measured 1736.7 ± 0.032 nM/1 mg (Figure 1), which is relatively lower than the standards for gallic acid, quercetin, ascorbic acid, rutin, trolox and BHT. However, the measured value of CLRE was comparable to the reference drug Silymarin which is 600.56 ± 0.003 nM/ 0.1 mg (Figure 1). This suggested that CLRE contained sufficient anti-oxidant efficacy to maintain the liver status quo.

### CLRE does not induce acute toxicity

**Figure 2:**
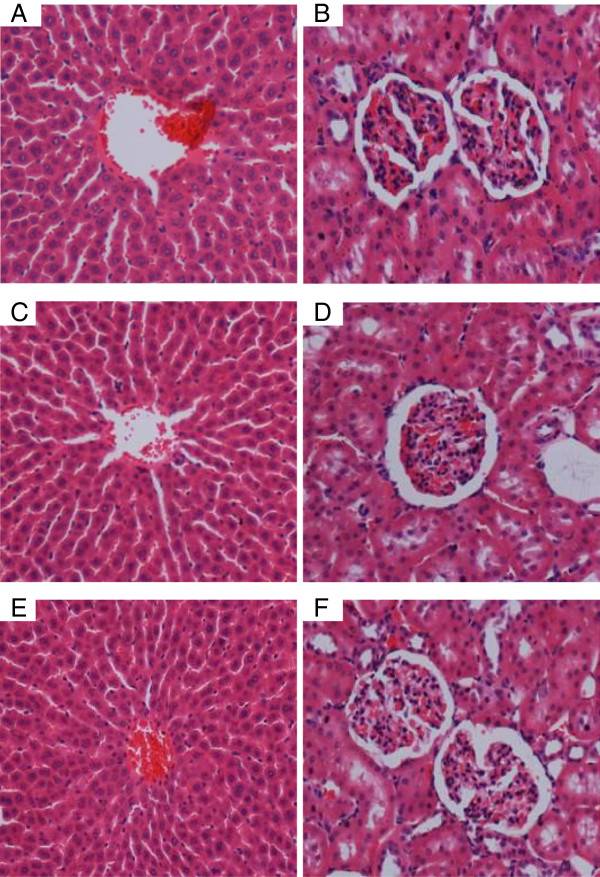
**Representative histological sections of liver and kidney in acute toxicity testing. **(**2A** and **2B**) Rats treated with 5 ml/kg vehicle (10% Tween-20). (**2C** and **2D**) Rats treated with 2 g/kg (5 ml/kg) CLRE. (**2E** and **2F**) Rats treated with 5 g/kg (5 ml/kg) CLRE. There was no significant difference in the structures of liver and kidney between treated and control groups (H&E stain ×20).

Following CLRE administration, all animals remained alive and did not manifest any visible toxicity at the doses used. Clinical observations and serum biochemistry did not show any significant differences between the control and the treated groups (Tables 1 and 2). Histopathology results of both liver and kidney (Figure 2) did not show any significant differences between controls and the treated groups.

**Table 1:** Effect of CLRE on renal function tests in rats

**Dose**	**Sodium**	**Potassium**	**Chloride**	**Urea**	**Creatinine**
	**(mM/L)**	**(mM/L)**	**(mM/L)**	**(mM/L)**	**(µM/L)**
**Vehicle (10% Tween-20)**	139.79 ± 1.34	4.87 ± 0.47	104.81 ± 1.42	4.69 ± 0.42	40.10 ± 2.63
**Low dose CLRE (2 g/kg)**	143.31 ± 2.11	5.14 ± 0.39	103.46 ± 2.04	4.97 ± 0.58	39.00 ± 2.71
**High dose CLRE (5 g/kg)**	140.67 ± 2.67	5.09 ± 0.40	103.70 ± 1.52	5.27 ± 0.52	38.82 ± 3.14

Values expressed as mean ± SEM. There are no significant differences between groups, significant value at *P*<0.05. CLRE: *C. longa* rhizomes ethanolic extract.

**Table 2:** Effect of CLRE on liver function tests in rats

**Dose**	**Total protein**	**Albumin**	**TB**	**AP**	**ALT**	**AST**	**GGT**
	**(g/L)**	**(g/L)**	**(µM/L)**	**(IU/L)**	**(IU/L)**	**(IU/L)**	**(IU/L)**
**Vehicle (10% Tween 20)**	68.33 ± 1.71	11.78 ± 0.76	1.74 ± 0.13	72.75 ± 5.53	37.65 ± 2.66	53.58 ± 5.20	4.50 ± 0.19
**Low dose CLRE (2 g/kg)**	71.17 ± 1.28	12.76 ± 0.58	2.15 ± 0.16	66.90 ± 5.40	39.17 ± 3.16	62.48 ± 2.63	4.08 ± 0..45
**High dose CLRE (5 g/kg)**	69.17 ± 1.85	12.26 ± 0.64	1.85 ± 0.46	70.08 ± 11.12	34.50 ± 2.91	55.17 ± 4.83	4.67 ± 0.33

Values expressed as mean ± SEM. There are no significant differences between groups. Significant value at *P*<0.05, *TB*: Total bilirubin; *AP*: Alkaline phosphatase; *ALT*: Alanine aminotransferase; *AST*: Aspartate aminotransferase; *GGT*: G-Glutamyl transferase; *CLRE*: *C. longa* rhizomes ethanolic extract.

### Effect of CLRE on liver cirrhosis

#### Body weight

Before sacrifice, the total body weight of each rat was measured. Rats from the normal control group (Group 1) followed a normal pattern of growth and attained a normal weight gain reaching 341.33 ± 6.184 g over 8 weeks. The cirrhosis rats (Group 2) suffered growth retardation and had a significantly (*P*<0.05) lower weight than other groups. When the body weights were factored in, rats from Group 2 measured the highest liver index. Silymarin-treated rats (Group 3) and the rats treated with a high dose (500 mg/kg) of CLRE extract (Group 5) attained weights equivalent to Group 1, the normal rats. Rats treated with the low dose (250 mg/kg) of CLRE extract (Group 4) gained more weight than those of Group 2 but not as much as those attained in Group 3 and 5 (Table 3). These findings suggested that high dose CLRE could be optimal since it was as effective as Silymarin in attenuating cirrhosis progression.

**Table 3:** Effect of CLRE on liver index measurements from rats at the end of 8 weeks study

**Treatment**	**Body weight (g)**	**Liver weight (g)**	**Liver weight × 100/body weight**
**Normal rats**	341.33 ± 6.18	8.43 ± 0.34	2.47 ± 0.06
**Cirrhosis rats**	216.83 ± 10.96	10.13 ± 0.54	4.70 ± 0.22**
**Silymarin-treated rats**	336 ± 9.187	9.12 ± 0.34	2.72 ± 0.14*
**Low dose CLRE-treated rats (250 mg/kg)**	249.17 ± 14.89	8.80 ± 0.29	3.58 ± 0.19*
**High dose CLRE-treated rats (500 mg/kg)**	351 ± 19.73	9.48 ± 0.19	2.74 ± 0.15*

Data are expressed as mean ± SEM. *P<0.001 compared with the cirrhosis control Group 2. **P<0.001 compared with normal control Group 1. CLRE: *C. longa* rhizomes ethanolic extract.

#### Specific liver markers and total protein, albumen and Bilirubin

**Figure 3:**
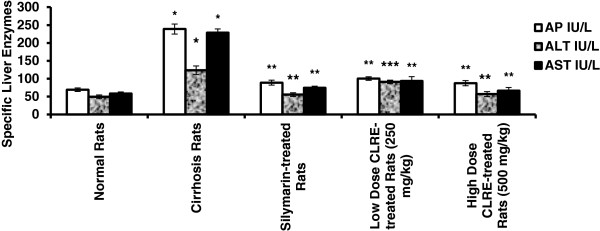
**Effect of CLRE on the plasma level of specific liver enzymes from rats at the end of 8 weeks study.** AP: alkaline phosphatase; ALT: alanine transferase; AST: aspartate transferase. Data were expressed as mean ± SEM. *P<0.001 compared with the normal control Group 1. **P<0.001 compared with cirrhosis control Group 2. ***P<0.05 compared with cirrhosis control Group 2.

**Figure 4:**
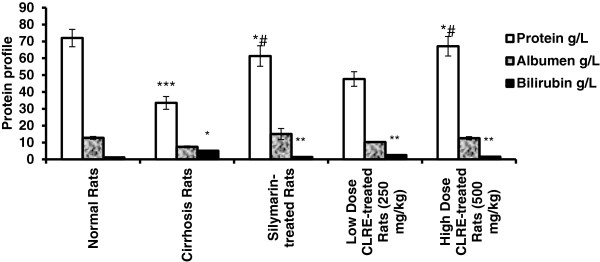
**Effect of CLRE on the total protein, albumen and bilirubin of rats at the end of 8 weeks study.** Data were expressed as mean ± SEM. *P<0.001 compared with normal control Group 1. **P<0.001 compared with cirrhosis control Group 2. ***P<0.01 compared with normal control Group1. *#*P<*0.01 compared with cirrhosis control Group 2.

**Figure 5:**
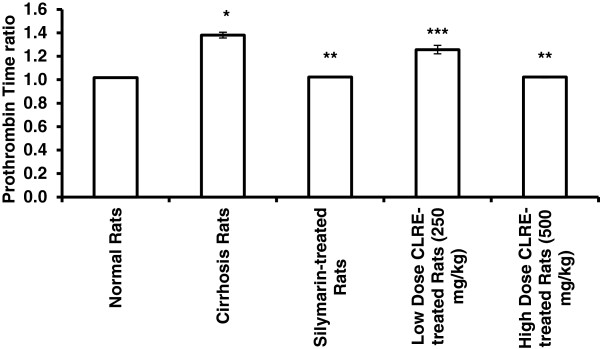
**Effect of CLRE on the prothrombin time ratio of rats at the end of 8 weeks study.** Data were expressed as mean ± SEM. *P<0.001 compared with the normal control Group 1. **P<0.001 compared with cirrhosis Group 2. ***P<0.01 compared with cirrhosis control Group 2.

The plasma levels of specific liver enzymes and protein profile was measured to determine the liver function of each rat (Figures 3, 4 and 5). The liver damage induced by TAA toxicity significantly (*P*<0.001) elevated the plasma level of specific liver enzymes (AP, ALT, AST, bilirubin and prothrombin time ratio) and significantly (*P*<0.001) lowered protein and albumen levels in the hepatotoxic rats of Group 2 compared with the other groups. The high dose CLRE-treated rats (Group 5) resulted in comparable biochemical marker readings to those of normal control Group 1 and Silymarin-treated Group 3, and better than those recorded from the low dose CLRE-treated rats (Group 4). These data demonstrated that the effects of toxicity induced by TAA on the liver function could be effectively counterbalanced by CLRE treatment.

#### Hepatic CYP2E1 levels

**Figure 6:**
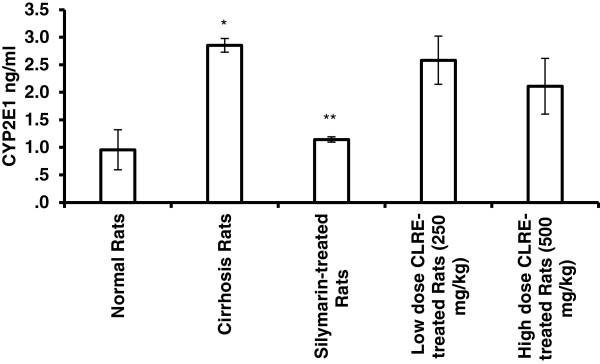
**Effect of CLRE on hepatic levels of CYP2E1 in rats at the end of 8 weeks study.** Data were expressed as mean ± SEM. *P<0.01 compared with the normal control Group 1. **P<0.05 compared with cirrhosis control Group 2. No significant difference was observed between the low dose CLRE-treated Group 4 and high dose CLRE-treated Group 5 when compared with cirrhosis Group 2.

As shown in Figure 6, animals from the cirrhosis Group 2 had significantly (*P*<0.001) higher levels of CYP2E1 compared with the normal Group 1 and Silymarin-treated Group 3. However, there was no difference between the low dose CLRE-treated animals of Group 4 and high dose CLRE-treated animals of Group 5 which had similar CYP2E1 levels, or between these groups and the cirrhosis Group 2.

#### Oxidative stress markers

Oxidative stress parameters (liver tissue homogenate MDA, nitrotyrosine, and urinary 8-OH-dG) are shown in Table 4. Generally, the cirrhosis rats treated with TAA only, had significantly higher levels of oxidative stress biomarkers (*P*<0.001) than the normal rats and the experimental treatment groups. Notably, the experimental rats treated with low dose and high dose CLRE had significantly lower levels (*P*< 0.001) of liver MDA and nitrotyrosine compared with the cirrhosis rats of Group 2. In addition, low and high dose treated rats had significantly lower levels (*P*<0.001) of urinary 8-OH-dG contents in comparison to the cirrhosis rats. Moreover, there were no significant differences in the tested oxidative stress biomarkers between CLRE-treated animals and Silymarin-treated animals. These results suggest that treatment with CLRE may protect hepatic cells from further damage during cirrhosis.

**Table 4:** Effect of CLRE on OHdG, Nitrotyrosine and MDA from rats at the end of 8 weeks study

**Treatment**	**8-OH-dG (ng/mL)**	**Nitrotyrosine (ng/mL)**	**MDA (nM/mg protein)**
**Normal rats**	2.17 ± 0.33	1.06 ± 0.07	2.17 ± 0.33
**Cirrhosis rats**	5.40 ± 0.34**	3.87 ± 0.13**	5.40 ± 0.34**
**Silymarin-treated Rats**	2.80 ± 0.15*	1.67 ± 0.07*	2.80 ± 0.15*
**Low Dose CLRE-treated rats (250 mg/kg)**	2.83 ± 0.33*	1.40 ± 0.20*	2.83 ± 0.33*
**High Dose CLRE-treated rats (500 mg/kg)**	2.37 ± 0.88*	1.33 ± 0.13*	2.37 ± 0.88*

Data are expressed as mean ± SEM. *P<0.001 compared with the cirrhosis control Group 2. **P<0.001 compared with normal control Group 1. CLRE: *C. longa* rhizomes ethanolic extract.

#### Hepatocellular antioxidant enzymes

**Figure 7:**
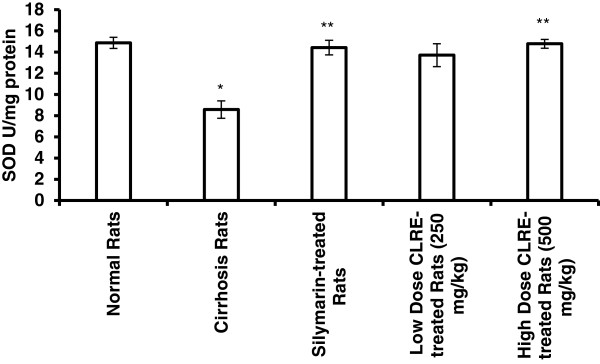
**Effect of CLRE on the levels of SOD enzyme in liver tissue homogenate at the end of 8 weeks study.** Data were expressed as mean ± SEM. *P<0.05 compared with the normal control Group 1. **P<0.05 compared with cirrhosis control Group 2.

**Figure 8:**
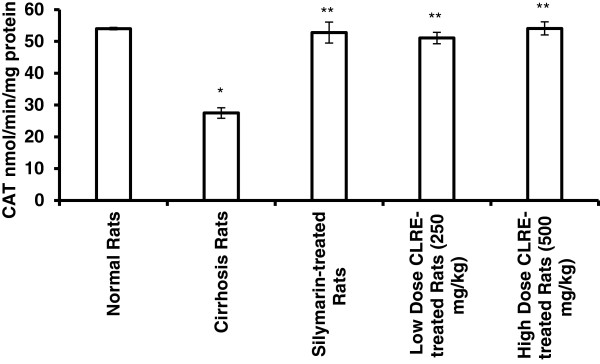
**Effect of CLRE on the levels of CAT in liver tissue homogenate at the end of 8 weeks study.** Data were expressed as mean ± SEM. *P<0.001 compared with the normal control Group 1. **P<0.001 compared with cirrhosis control Group 2.

The loss of hepatocytes in the cirrhotic livers of animals was indirectly analyzed by the activity of the antioxidant enzymes (SOD and CAT). SOD and CAT results were similar to that of the oxidative stress biomarkers (Figures 7 and 8), but inversely, so the values of SOD and CAT in the cirrhotic rats were lower than in the normal rats. These results indicated the occurrence of severe damage in the cells of cirrhotic livers. Treating the cirrhotic animals with low and high dose CLRE significantly (*P*<0.05) increased the levels of SOD and CAT and induced the survival of hepatocytes. These results collectively supported the suggestion that treatment with CLRE could provide a favorable host environment for protecting the hepatocytes from progressive damage.

#### Cytokine assessment

**Figure 9:**
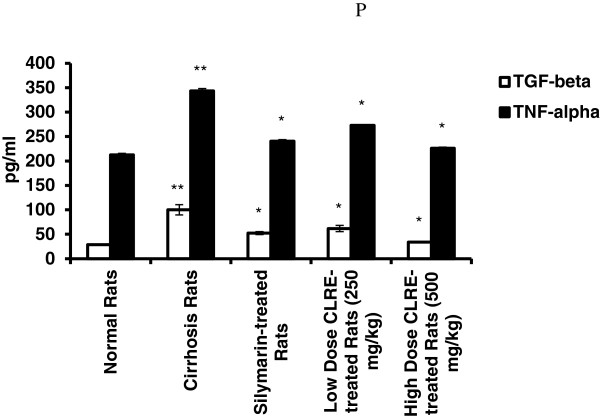
**Effect of CLRE on the serum levels of cytokines at the end of 8 weeks study.** Data were expressed as mean ± SEM. *P<0.001compared with the cirrhosis control Group 2. **P<0.001 compared with normal control Group 1.

The serum levels of the cytokines TGF-β and TNF-α from samples collected from all sacrificed rats are shown in Figure 9. TGF-β1 and TNF-α levels were significantly elevated (*P*<0.001) in serum samples from the cirrhosis Group 2 (100.11 ± 10.67 and 343.41 ± 4.66 pg/mL, respectively) compared with all other groups. Administration of CLRE to animals reduced the serum levels of the fibrogenic factor TGF-β1 in the low dose CLRE-treated rats (61.72 ± 6.27 pg/mL) and in the high dose CLRE-treated rats (34.11 ± 0.84 pg/mL). In addition, the serum levels of the inflammatory mediator TNF-α decreased in the low dose CLRE-treated group 4 (272.73 ± 1.61 pg/mL) and in the high dose CLRE-treated Group 5 (226.30 ± 2.01 pg/mL). Levels of TGF-β1 and TNF-α from the high dose CLRE-treated rats approached the values from the Silymarin-treated group (52.44 ± 2.96 and 240.54 ± 4.66 pg/mL, respectively) compared with higher values in the low dose CLRE-treated rats.

#### Pro-apoptotic Bax and anti-apoptotic Bcl-2 assessment

**Figure 10:**
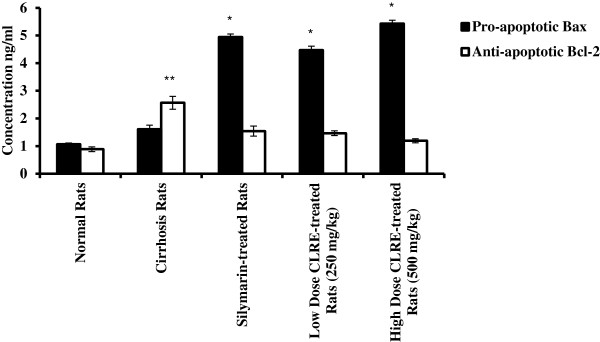
**Effect of CLRE on the serum levels of the pro-apoptotic Bax and anti-apoptotic Bcl-2 at the end of 8 weeks study.** Data were expressed as mean ± SEM. *P<0.001compared with the cirrhosis control Group 2^. **^P<0.05 compared with normal control Group 1.

**Figure 11:**
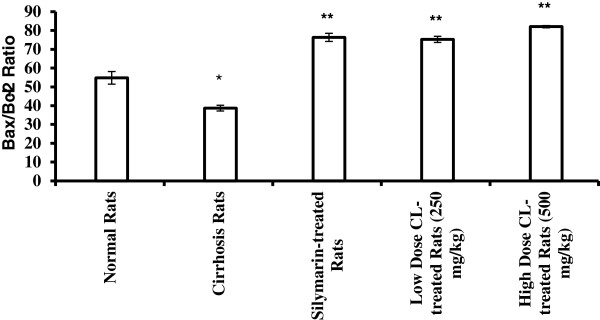
**The ratio between pro-apoptotic protein Bax and anti-apoptotic protein Bcl-2.** Data were expressed as mean ± SEM. *P<0.01compared with normal control Group 1. **P<0.001 compared with cirrhosis control Group.

The level of the pro-apoptotic protein Bax and the anti-apoptotic protein Bcl-2 in the rat sea and the ratio of Bax/Bcl-2 are shown in Figures 10 and 11. Results of Bax showed no significance differences between the cirrhosis group 2 and the normal group 1. On the other hand, there was significant increase (*P*<0.001) in the level of Bax in silymarin-treated Group 3, low dose CLRE-treated Group 4 and high dose CLRE-treated group 5 (4.98 ± 0.11, 4.47 ± 0.15 and 5.43 ± 0.12 ng/mL respectively) compared to the cirrhosis group 2. The level of anti-apoptotic protein Bcl-2 showed significant increase (*P*<0.05) in the cirrhosis group 2 compared with the normal group 1 (2.57 ± 0.23 and 0.89 ± 0.09 ng/mL respectively), whereas no significance differences were observed between the high dose and the low dose treated groups when compared with the cirrhosis group 2 indicating enhanced apoptosis in silymarin and CLRE- treated groups as confirmed by the ratio Bax/Bcl-2 in Figure 11.

#### Gross anatomy and histopathology

**Figure 12:**
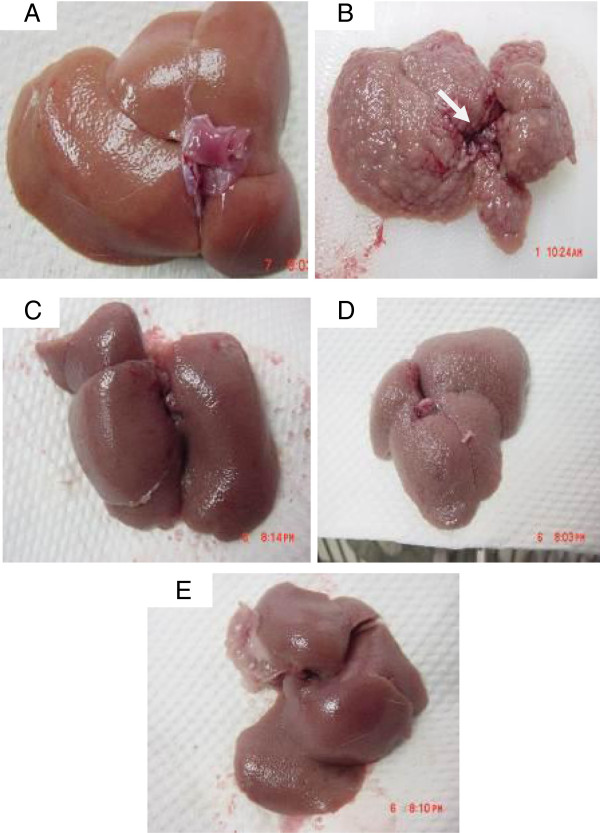
**Representative images showing the macroscopic appearances of livers sampled from rats in different experimental groups. **(**12A**) The liver of a control rat exhibiting a regular smooth surface. (**12B**) The liver of a hepatotoxic rat depicting numerous irregular whitish micro- and macronodules on its surface and a large area of ductular cholangiocellular proliferation (arrow) embedded within fibrotic areas. (**12C**) The liver of a hepatoprotected rat treated with Silymarin showing a normal smooth surface. (**12D**) The liver of a rat treated with low dose CLRE with a nearly smooth surface with few granules (arrow head). (**12E**) The liver of a rat treated with high dose CLRE with a normal smooth surface.

**Figure 13:**
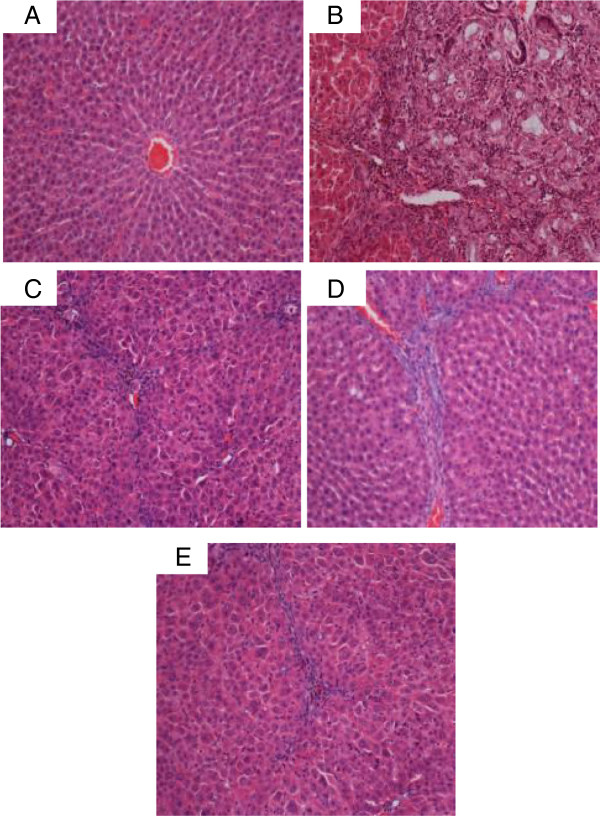
**Examples of representative histopathological sections from livers sampled from rats in different experimental groups. **(**13A**) Normal histological structure and architecture were seen in livers from the control rats. (**13B**) Severe structural damage, formation of pseudolobules with thick fibrotic septa and necrotic areas were present in the livers of hepatotoxic rats. (**13C**) Mild inflammation but no fibrotic septae were observed in the liver of the hepatoprotective rat treated with Silymarin. (**13D**) Partially preserved hepatocytes and architecture with small area of necrosis and fibrotic septa were observed in the livers of rats treated with low dose CLRE. (**13E**) Partially preserved hepatocytes and architecture with small areas of mild necrosis were observed in the liver of rats treated with high dose CLRE. (H&E stain, original magnification ×20).

The gross appearances of the liver samples and microscopic assessment (H&E staining) of their sections in the experimental Groups 1–5 are shown in Figures 12 and 13. The gross appearance of livers from the normal rats in Group 1 (Figure 12A) appeared reddish with smooth surfaces and without signs of nodules, and histology showed normal architecture (Figure 13A). Livers from the cirrhotic rats of Group 2 appeared congested with numerous micro- and macro-nodules (Figure 12B). The normal liver architecture was lost and replaced by regenerating nodules that were separated by fibrous septae extending from the central vein to the portal triad (Figure 13B) and accompanied by intensive proliferation of the bile duct together with invasive inflammatory cells. In addition, thick purple bundles of collagen fibers appeared in the cirrhotic nodules. The livers of Silymarin-treated Group 3 (Figures 12C and 13C) and the high dose CLRE-treated Group 5 (Figures 12E and 13E) showed minor- micro-nodules with less fibrous septae and more extension of normal hepatic parenchyma. In contrast, the livers of the low dose CLRE-treated Group 4 (Figures 12D and 13D) showed less fibrotic macro-nodules than those of the Silymarin-treated Group 3, but the improvements were not great as those seen in Groups 3 and 5. These visual evaluations provide further independent confirmation that CLRE treatment effectively protected the liver from further cirrhosis in a dose dependent manner.

#### Masson’s Trichrome staining

**Figure 14:**
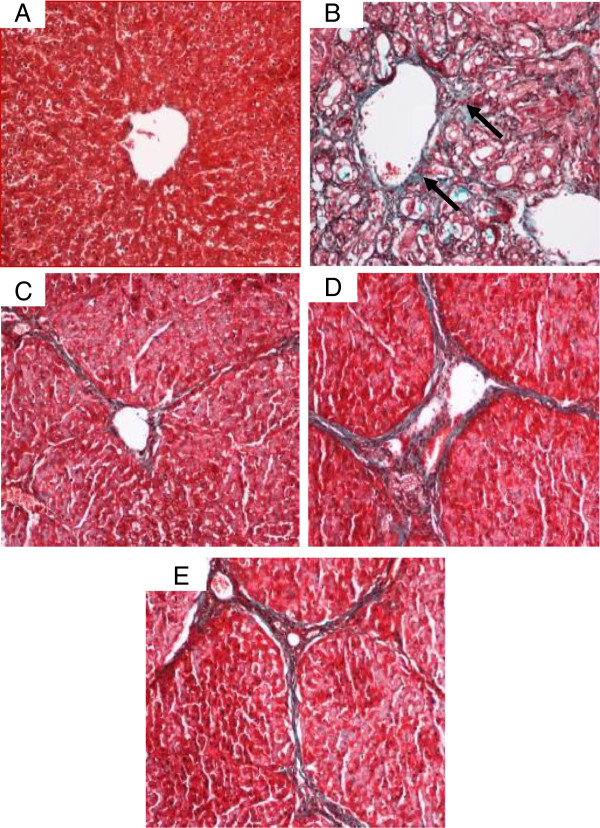
**Masson’s Trichrome staining of representative livers sampled from rats in different experimental groups. **(**14A**) Normal liver structure without signs of collagen deposition in livers from control rats. (**14B**) Severe collagen deposition (arrow) and severe fibrosis were seen in the livers from cirrhosis rats. (**14C**) Minimal collagen deposition in the liver of the hepatoprotective rat treated with Silymarin. (**14D**) Moderated collagen deposition and moderate congestion around the central vein in the livers of rats treated with low dose CLRE. (**14E**) Mild collagen deposition was observed in the livers of rats treated with high dose CLRE. (Original magnification ×20).

The degree of fibrosis determined by Masson’s trichrome staining of the liver sections from all of the treated groups is illustrated in Figure 14. Liver sections from the normal rats (Figure 14A) appeared normal without signs of collagen deposition. Liver sections from the cirrhosis rats of Group 2 revealed increased deposition of collagen fibers around the congested central vein indicating severe fibrosis (Figure 14B). Liver tissues from Silymarin-treated Group 3 (Figure 14C) showed minimal collagen deposition indicating minimal fibrosis. Livers from rats treated with low dose CLRE showed moderate deposition of collagen fibers and moderate congestion around the central vein (Figure 14D), while those from rats treated with high dose CLRE showed mild collagen deposition and mild congestion around the central vein (Figure 12E). This evaluation of the degree of fibrosis confirms the previous findings that CLRE treatment protected the livers of animals from progressive fibrosis.

#### Immunohistochemistry of Bax, Bcl2 and PCNA

**Figure 15:**
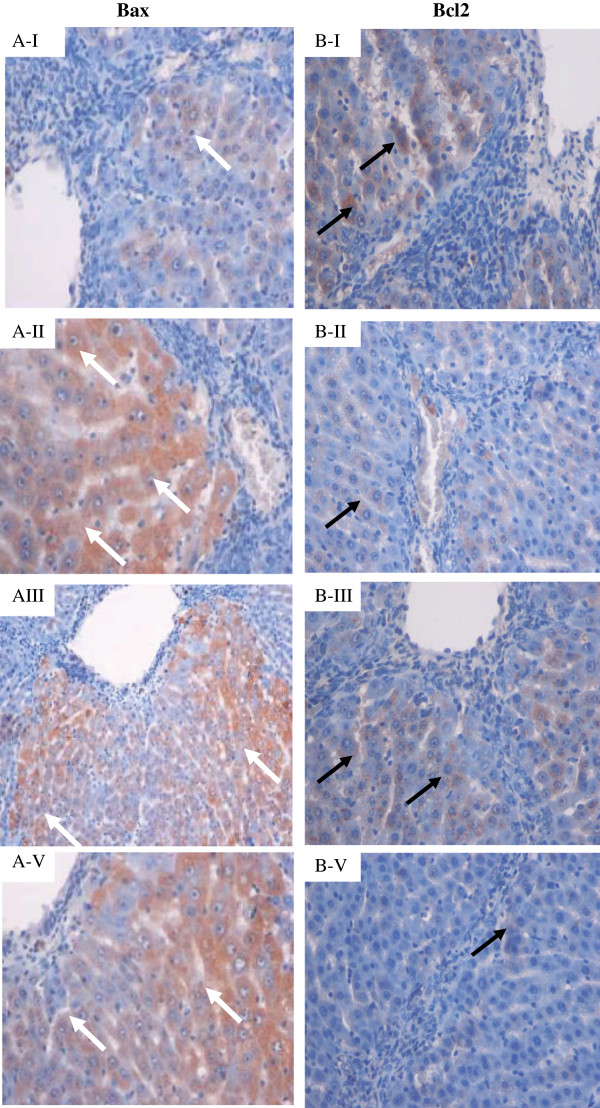
**Bax and Bcl2 staining of representative livers sampled from rats in different experimental groups. **(**A-I**) Less apoptosis indicated by few Bax-positive hepatocytes (white arrow) and (B-I) more Bcl2-positive hepatocytes (black arrow) in liver tissues from cirrhosis rats. (**A-II**) High numbers of Bax-positive hepatocytes (White arrow) and (B-II) very less Bcl2-positive hepatocytes (black arrow) in the livers from hepatoprotected rats treated with Silymarin. (**A-III**) Moderate apoptosis as indicated by moderate Bax staining (white arrow) and (**B-III**) moderate Bcl2 staining (black arrow) indicating moderate apoptosis in the livers from rats treated with low dose CLRE. (A-V) High numbers of Bax-positive cells (white arrow) with severe apoptosis and (B-V) very less Bcl2-positive hepatocytes (black arrow) were observed in the liver of rats treated with high dose CLRE. (Original magnification ×40).

**Figure 16:**
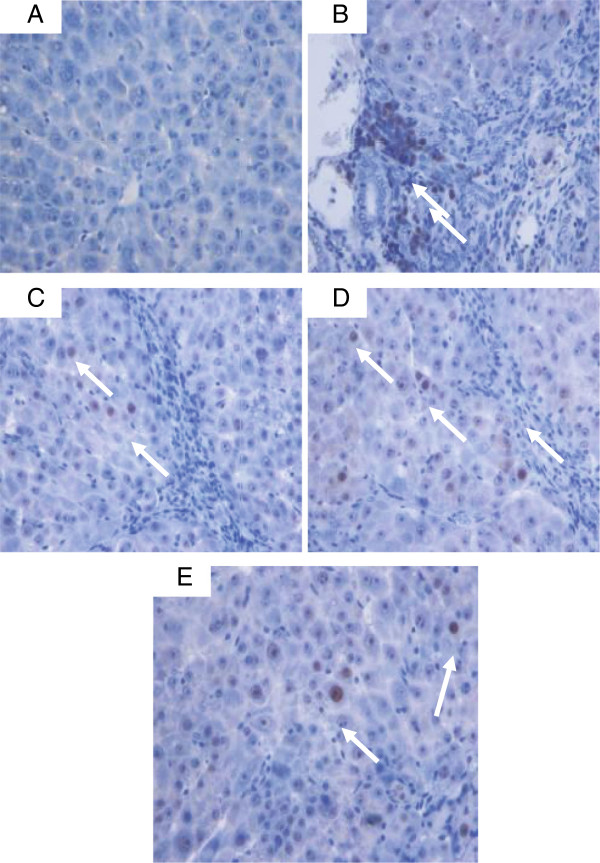
**PCNA staining of livers sampled from rats in different experimental groups. **(**16A**) Normal livers without signs of PCNA expression in hepatocytes from control rats. (**16B**) Severe fibrosis with greater PCNA expression in the hepatocytes from cirrhosis rats. (**16C**) Less PCNA-stained hepatocytes (arrow) indicating less hepatocyte regeneration in the liver of hepatoprotected rats treated with Silymarin. (**16D**) Moderate hepatocyte regeneration as indicated by moderate PCNA staining (arrow) in the liver of rats treated with low dose CLRE. (**16E**) Minor PCNA expression (arrow) with few regenerative hepatocytes was observed in the liver of rats treated with high dose CLRE. (Original magnification ×40).

Bax, Bcl2 and PCNA staining of hepatocytes from the livers of all experimental groups are shown in Figures 15A, 15B and 16. Hepatocytes of liver tissues from the cirrhosis rats of Group 2 showed down-regulation of Bax staining (Figure 15A-15I) with up-regulation of Bcl2-positive hepatocytes (Figure 15B-15I) and more PCNA staining (Figure 16B) indicating severe damage with increased number of necrotic cells than their apoptosis. Hepatocytes from Silymarin-treated rats of Group 3 showed up-regulated Bax expression (Figure 15A-II), down-regulated Bcl2 expression (Figure 15B-II) and, a few PCNA staining (Figure 16C) indicating lower levels of proliferation of necrotized hepatocytes and enhanced apoptosis. Liver tissues treated with low dose CLRE and high dose CLRE induced hepatocyte apoptosis as indicated by up-regulated Bax expression (Figures 15A-III and 15A-V), down-regulated Bcl2 (Figures 15B-III and 15B-V) and down-regulated necrotized hepatocytes’ proliferation as indicated by reduced PCNA staining (Figures 16D and 16E). These findings supported the idea that CLRE extract might induce hepatoprotective activity against progressive liver damage by increasing apoptosis of damaged hepatocytes and ameliorating their proliferation.

## Discussion

Prescription drugs with side effects have become widely used in modern life and as a result, liver cirrhosis has become a serious health problem. Consequently, the current study focused on finding new therapeutic solutions to minimize liver damage [31]. Natural products, especially plants in folk medicine with an anecdotal history of positive effects against liver diseases or other organs, are considered an alternative therapeutic approach [32]. In the present research, the ethanol extract of *C. longa* rhizomes was examined as a promising therapy for treating liver cirrhosis. This study evaluated the toxicity of CLRE along with the clinical biochemistry values, which confirmed by the biochemical results (Tables 1 and 2). In addition, histological examination showed no significant pathological abnormalities in both the liver and the kidney, even at high doses of 5 g/kg (Figure 2). The hepatoprotective effects of CLRE on the development of liver cirrhosis, induced by prolonged exposure to TAA (200 mg/kg ) were assessed though this study. The protocol induced cirrhosis with similar pathology and etiology pattern to the human liver cirrhosis with the same biochemical values for typical human cirrhosis markers [33]. The results were reconfirmed quantitatively by measurement of the liver index of the cirrhotic animals (Table 3), the biochemical imbalances in the liver markers (Figures 3 and 5) and the altered total protein content, albumen and bilirubin levels (Figure 4). A marked reduction of plasma total protein levels were observed in the cirrhosis control Group 2 compared with the normal healthy animals of Group 1 (Figure 4), as described in other TAA intoxication models [34]. Hepatic factors (AP, ALT, and AST) were significantly increased in the cirrhosis control rats, as previously described [34]. CLRE-treatment caused significant recovery of these enzymatic activities (Figure 3). Parallel findings were also previously reported [35].

TAA has been used to induce hepatotoxicity in the experimental animals to produce various grades of liver damage including nodular cirrhosis, liver cell proliferation, production of pseudolobules, and parenchymal cell necrosis [36]. It is a potent hepatotoxic agent metabolized by CYP2E1 enzymes present in liver microsomes and is converted to a toxic reactive intermediate called thioacetamide by oxidation [37]. Here, we measured the levels of CYP2E1 in the liver tissues of all animals and found that CLRE extract administration was not as effective as Silymarin (Figure 6) in terms of hepatic CYP2E1 inhibition and attenuating drug-induced hepatotoxicity [38]. Parallel findings reported that curcumin, the active ingredient of *C. longa* rhizomes and constitutes 2.5-6% of the plant rhizome constituents [39] had no significant effect on CYP2E1 [30,40].

The development of liver cirrhosis by TAA was reported to be multifaceted involving multiple mechanisms [41]. For instance, TAA induces hepatocyte damage via its metabolite, TASO_2_, which covalently binds to macromolecules of hepatocytes causing DNA damage, protein oxidation and lipid peroxidation of the cell membrane biomolecules [25,42]. In the present study, we evaluated the oxidative stress markers and observed that the level of damage to liver cells due to oxidative stress was very high in the cirrhosis rats of Group 2 as indicated by high levels of urine 8-OH-dG, nitrotyrosine and MDA (Table 4). However, the levels in low-dose and high dose CLRE-treated rats were encouragingly close to that of Silymarin-treated rats, supporting previous studies on the protective effect of *C. longa* against oxidative stress by down-regulation of ROS [43] by inhibiting DNA damage and attenuating protein and lipid oxidation of hepatocytes as indicated by low levels of urine 8-OH-dG, nitrotyrosine and MDA respectively in CLRE-treated animals.

Reduced hepatic antioxidant functions have also been suggested to be one mechanism of TAA-induced hepatotoxicity [44]. Our results revealed that administration of CLRE to the cirrhotic rats significantly alleviated the TAA-suppressive effect on antioxidant enzymes SOD and CAT by maintaining the activity of these enzymes at higher levels (Figures 7 and 8). Optimizing the level of hepatocellular antioxidant enzymes led to removal of oxidative stress by scavenging the free radicals resulting from TAA-toxicity. Antioxidant activity of CLRE may be attributed to the antioxidant properties of phenol compound constituents which constitute 3-15% of rhizomes [45] and that TPC is equivalent to gallic acid (517.54 ± 0.049 mg GAE/mg extract). Toxins target metabolically active hepatocytes [46] leading to hepatocyte dysfunction and the release of ROS, and fibrogenic and inflammatory mediators. Several studies have suggested that part of hepatocellular injury induced by TAA is mediated through oxidative stress caused by the action of cytokines through lipid peroxidation [47]. The free radicals resulting from TAA metabolism may activated myofibroblasts, that secrete fibrinogen and growth factors [48]. TGF-β1, a prominent profibrogenic cytokine with antiproliferative effects that can up-regulate the deposition of ECM [49], was present at high levels in the cirrhosis rats of Group 2 compared with the other groups (Figure 9). In addition, the pro-inflammatory cytokine TNF-α [50] was elevated in the cirrhosis rats indicating a high inflammatory state in the cirrhotic liver. Low or high dose CLRE administration to the rats reduced the high levels of cytokines in their sera, supporting previous reports on the inhibitory effects of curcumin on the transcription of nuclear factor NF-κB binding activity, and TNF-α [30] and TGF-β1 expression [51].

Upon liver injury, hepatic stellate cells acquire a highly proliferative index producing fibrillar collagen within the injured liver [52]. Our biochemical findings were supported by the gross and histopathological examinations of the rat liver tissues (Figures 10 and 11) showing that livers from CLRE-treated rats had nearly normal liver architecture. In addition, Masson’s Trichrome staining showed significant improvement in collagen synthesis upon administration of CLRE to the rats treated with TAA. This was probably due to the inhibitory effect of CLRE on hepatic stellate cell activation. These findings confirmed previous studies, demonstrating the attenuating effect of curcumin against liver fibrosis by inhibiting HSC activity [53]. Curcumin, the most common antioxidant constituent of *Curcuma longa* rhizome extract, was reported to enhance apoptosis of damaged hepatocytes which might be the protective mechanism whereby curcumin down-regulated inflammatory effects and fibrogenesis of the liver [53].

A number of studies have focused on the molecular regulation of apoptosis. Over expression of the pro-apoptotic proteins Fas, FasL and Bax were reported in chronic hepatitis [54]. Toxicity induced by TAA was found to be accompanied by elevation in Bax protein levels and reduction in the anti-apoptotic protein Bcl2 and its translocation into the mitochondria, causing apoptosis [55]. In the current study, we observed significant increase in the serum level of Bax protein and decrease in Bcl-2 protein in silymarin-treated and CLRE- treated animals compared with the cirrhosis group animals. This was confirmed by the ratio Bax/Bcl-2 which was high in the treated groups compared with the cirrhosis group and the large number of Bax positive-stained hepatocytes together with few Bcl2 positive-stained hepatocytes both doses of CLRE-treated animals, and in Silymarin-treated animals compared with the cirrhosis Group (Figures 10, 11, 13A and 13B) indicating the susceptibility of these cells to apoptosis and the role of curcuminoids in inducing apoptosis [53]. Furthermore, those animals on daily feeding with CLRE along with TAA injections thrice weekly for 8 weeks attenuated hepatocyte proliferation and regeneration as indicated by a significant decrease in PCNA positive-stained cells in the liver sections from the low dose and high dose-treated groups similar to that in the Silymarin-treated group (Figure 14) [56]. These results were consistent with previous reports that curcumin the active ingredient of CLRE extract had inhibitory effect on hepatocyte proliferation [53]. Treating the animals with CLRE extract inhibited the necrotic effect due to thioacetamide administration by modifying necrosis into apoptosis, which might be through cytochrome release from mitochondria and caspase activation [57]. This modification *in vivo* would scale down the release of inflammatory mediators that would prevent progressive live damage. The ethanolic extract of *C. longa* rhizomes showed a significant hepatoprotective effect when orally administrated in doses of 250 mg/kg and 500 mg/kg, and the protective effect was dose-dependent. The main constituents of CLRE extract are the flavonoid curcumin and various volatile oils, including tumerone, atlantone, and zingiberene. The hepatoprotective effects of turmeric and curcumin might be due to direct antioxidant and free-radical scavenging mechanisms, as well as the ability to indirectly augment glutathione levels, thereby aiding in hepatic detoxification [58]. The volatile oils and curcumin of *C. longa* exhibit potent anti-inflammatory effects [59].

## Conclusion

In conclusion, our results demonstrated that the progression of TAA-induced liver cirrhosis could be prevented or reduced using the ethanol extract of *C. longa* rhizomes. The plant natural extract exerted its hepatoprotective effect by preventing the harmful cascade of events induced by TAA toxicity. This hepatoprotective capability of CLRE preserved the liver’s status quo in terms of its properties, functions and structure against toxins, and warranted further study to explore its pharmacologic potential in treating liver cirrhosis. In addition, Curcumin might be predominantly responsible for the hepatoprotective effect of CLRE rhizome extract. These findings would encourage further studies on the pharmacological significance of using plant extracts as alternative medicines for treating liver cirrhosis.

## Competing interests

No competing interests of either a financial or non-financial nature.

## Authors’ contributions

SMS: Designing the research project, collection, analysis, and interpretation of data; writing of the manuscript and the decision to submit the manuscript for publication. MAA: Designing the research project; Animal experiment and the decision to submit the manuscript for publication. AS: Interpretation of data and writing of the manuscript. SS: Collection of data and revision of the manuscript. SG: Interpretation of data. SI: Involved in the decision to submit the manuscript. All authors read and approved the final manuscript.

## Pre-publication history

The pre-publication history for this paper can be accessed here:

http://www.biomedcentral.com/1472-6882/13/56/prepub
